# What Remains to Be Discovered in Schizophrenia Therapeutics: Contributions by Advancing the Molecular Mechanisms of Drugs for Psychosis and Schizophrenia

**DOI:** 10.3390/biom14080906

**Published:** 2024-07-25

**Authors:** Christoph U. Correll, Massimo Tusconi, Mauro Giovanni Carta, Serdar M. Dursun

**Affiliations:** 1 Department of Psychiatry, Zucker Hillside Hospital, Northwell Health, Glen Oaks, NY 10128, USA; ccorrell@northwell.edu; 2 Department of Psychiatry and Molecular Medicine, Donald and Barbara Zucker School of Medicine at Hofstra/Northwell, Hempstead, NY 11549, USA; 3 Department of Child and Adolescent Psychiatry, Charité—Universitätsmedizin Berlin, 10117 Berlin, Germany; 4 University Hospital of Cagliari, 09123 Cagliari, Italy; 5 Department of Medical Sciences and Public Health, University of Cagliari, 09124 Cagliari, Italy; maurogcarta@gmail.com; 6 Neurochemical Research Unit, Department of Psychiatry, University of Alberta, Edmonton, AB T6G 2G5, Canada; dursun@ualberta.ca

**Keywords:** schizophrenia, treatment, animal models, receptors, biomarkers

## Abstract

Schizophrenia is a frequently debilitating and complex mental disorder affecting approximately 1% of the global population, characterized by symptoms such as hallucinations, delusions, disorganized thoughts and behaviors, cognitive dysfunction, and negative symptoms. Traditional treatment has centered on postsynaptic dopamine antagonists, commonly known as antipsychotic drugs, which aim to alleviate symptoms and improve functioning and the quality of life. Despite the availability of these medications, significant challenges remain in schizophrenia therapeutics, including incomplete symptom relief, treatment resistance, and medication side effects. This opinion article explores advancements in schizophrenia treatment, emphasizing molecular mechanisms, novel drug targets, and innovative delivery methods. One promising approach is novel strategies that target neural networks and circuits rather than single neurotransmitters, acknowledging the complexity of brain region interconnections involved in schizophrenia. Another promising approach is the development of biased agonists, which selectively activate specific signaling pathways downstream of receptors, offering potential for more precise pharmacological interventions with fewer side effects. The concept of molecular polypharmacy, where a single drug targets multiple molecular pathways, is exemplified by KarXT, a novel drug combining xanomeline and trospium to address both psychosis and cognitive dysfunction. This approach represents a comprehensive strategy for schizophrenia treatment, potentially improving outcomes for patients. In conclusion, advancing the molecular understanding of schizophrenia and exploring innovative therapeutic strategies hold promise for addressing the unmet needs in schizophrenia treatment, aiming for more effective and tailored interventions. Future research should focus on these novel approaches to achieve better clinical outcomes and improve the functional level and quality of life for individuals with schizophrenia.

## 1. Introduction

Schizophrenia is a debilitating and complex mental disorder that affects approximately 1% of the global population [1]. Characterized by a wide range of symptoms, including hallucinations, delusions, cognitive dysfunction, and negative symptoms [2,3,4], schizophrenia poses significant challenges to patients, their families, and the healthcare system [5,6]. For decades, postsynaptic dopamine antagonists, commonly also known as “antipsychotic” drugs, have been the cornerstone of schizophrenia treatment, aiming to alleviate distressing symptoms and improve the quality of life for affected individuals [7]. However, despite the availability of these medications, there are still significant gaps and challenges in schizophrenia therapeutics [8]. This article delves into the evolving landscape of the development of drugs for psychosis and schizophrenia [9], focusing on the molecular mechanisms, novel drug targets, and innovative delivery methods that hold promise in addressing these challenges.

## 2. Schizophrenia Treatment Gaps and Challenges

### Terminology: From “Anti-psychotic” to “Drugs for Psychosis”

The terminology used to describe medications for schizophrenia has evolved over time [10,11]. The term “antipsychotic” has been traditionally used to describe drugs that alleviate psychotic symptoms, such as hallucinations and delusions [12]. While this term accurately reflects the primary action of these drugs, it can be limiting in capturing the broader scope of their effects. To address this limitation and emphasize the underlying mechanisms [13] (Table 1), a more neuroscientific nomenclature has been proposed: “drugs for psychosis” [14,15].

This shift in terminology highlights the fact that these medications [16] primarily target the neural mechanisms underlying psychosis, rather than simply treating the symptoms [17,18,19]. Understanding the molecular pathways and neural circuits involved in psychosis is crucial for the development of more effective treatments [20].

## 3. “Anti-psychotic” vs. Anti-Schizophrenia Treatments

Schizophrenia is not a monolithic condition solely characterized by psychosis [21]. It encompasses a spectrum of symptoms, including negative symptoms [22] (such as anhedonia and social withdrawal) [23], cognitive dysfunction (such as impaired memory and executive function), and affective symptoms (such as depression and anxiety) [24]. Traditional postsynaptic dopamine antagonist “antipsychotic” drugs primarily target psychosis-related symptoms by modulating neurotransmitter systems, particularly the dopamine system [25,26] (Table 1). However, there is a growing recognition of the need for treatments that address the broader spectrum of schizophrenia symptoms [27].

The term “anti-schizophrenia treatments” is emerging to encompass therapies that target multiple symptom domains, including positive, negative, cognitive, and affective symptoms. These treatments aim to provide comprehensive relief for individuals with schizophrenia and improve their overall functioning and quality of life [28,29,30].

## 4. Problems and Opportunities of Animal Models and Drug Effects

### Animal Models of Schizophrenia

One of the challenges in schizophrenia research lies in the translation of findings from animal models to human patients [31]. Animal models, typically rodents, have been instrumental in understanding the neurobiology of schizophrenia and testing potential drug candidates [32,33]. However, these models have limitations in capturing the complexity of the human condition [34,35]. Schizophrenia is a uniquely human disorder, and animal models can only simulate certain aspects of the disease [36,37]. Thus, while animal models can provide valuable insights into the neurobiology and potential therapeutic interventions [38], they cannot fully replicate the intricate interplay of genetic, environmental, and developmental factors that contribute to schizophrenia in humans [39,40,41] (Table 1).

Animal models are essential for understanding the neurobiological mechanisms of schizophrenia and developing new therapies [37]. Among the most commonly used models are those based on the pharmacologic stimulation of “psychosis-like” disorganized cognitive or behavioral symptoms with dopamine agonists or NMDA antagonists and genetic manipulation, such as mice with mutations in the DISC1, NRG1, and DTNBP1 genes, which replicate some neurophysiological and behavioral characteristics of schizophrenia [41]. Other models include prenatal exposure to infections or stress, mimicking environmental risk factors associated with the disease [42]. Despite their utility, these models have significant limitations. The complex cognitive and symptomatic features of human schizophrenia, such as hallucinations and delusions, cannot be fully replicated in animals [43]. Moreover, differences between the human brain and those of laboratory animals make it challenging to translate findings directly to clinical settings [44]. These limitations highlight the need to develop more sophisticated models and to combine data from animal studies with human research for a more comprehensive understanding of schizophrenia.

## 5. “Anti-psychotic” Drug Effects in Animal Models

Understanding the effects of drugs for psychosis in animal models is crucial for drug development. These models help researchers assess the efficacy and safety of potential medications before advancing to human clinical trials. However, translating the effects observed in animals to human outcomes can be challenging [45]. Researchers face the dilemma of selecting appropriate animal proxies for schizophrenia symptoms. The choice of behavioral assays and endpoints can significantly impact the interpretation of drug effects. Innovative approaches, such as “smart box screening”, are being explored to address these challenges. Smart box screening involves the use [46] of sophisticated behavioral assays and monitoring systems to assess a drug’s impact on specific receptor systems and neural circuits [47].

## 6. Established and Emerging Drug Discovery Approaches: Hypothesis-Free Rapid Screening

To address the gaps in schizophrenia therapeutics, researchers are exploring novel drug discovery approaches [48]. The hypothesis-free rapid screening of compounds is one such approach. This method involves systematically testing a wide range of compounds for their effects on specific receptor systems and neural circuitry targets [49]. By using a hypothesis-free rapid screening approach, researchers can identify promising candidates without preconceived notions about their mechanisms of action. This strategy can accelerate drug discovery by identifying compounds that exhibit the desired effects on psychosis-related neural circuits.

## 7. Reducing Preclinical to Clinical Translation Failures

One of the critical challenges in drug development is the failure to translate promising preclinical findings into successful clinical outcomes [50]. To mitigate these translation failures, researchers are working to establish robust predictive biomarkers and improve the validity of animal models [51,52,53,54,55,56]. The development of reliable biomarkers that correlate with treatment response in schizophrenia patients can aid in patient stratification and the early identification of responders and non-responders [57,58]. This approach can reduce the variability observed in clinical trials and increase the likelihood of success.

## 8. Lessons Learned from “Anti-psychotic” Drug Development Failures

The history of the development of drugs for psychosis and schizophrenia is marked by both successes and failures. Understanding the reasons behind the failures can guide future research efforts. Some common pitfalls in drug development include the following [9,59]:

1. Lack of specificity: Many drugs currently approved for schizophrenia target multiple receptors, leading to a range of side effects. Identifying more selective drug candidates can improve tolerability [60].
2. Overemphasis on the dopamine system: While dopamine dysregulation plays a role in psychosis, it is not the sole contributor to schizophrenia. Expanding the focus to other neurotransmitter systems is essential [61].
3. Neglect of cognitive and negative symptoms: Traditional “antipsychotic” drugs as studied in people with schizophrenia primarily target positive symptoms (hallucinations and delusions) but often have limited efficacy for cognitive and negative symptoms. Developing treatments for these domains is crucial [62].

## 9. Beyond Neurotransmitter–Receptor Dyads: Intracellular Signaling as Antipsychotic Targets

### Biased Agonism as an Example of Complexity

Schizophrenia’s pathophysiology is not solely reliant on neurotransmitter–receptor interactions. Intracellular signaling pathways within neurons play a crucial role in shaping the disease’s manifestations [63]. One intriguing concept is biased agonism, which refers to the selective activation of specific signaling pathways downstream of a receptor [64,65]. Biased agonists can modulate intracellular signaling in unique ways, offering the potential for more precise and nuanced pharmacological interventions [66,67,68,69,70]. By targeting specific intracellular pathways associated with schizophrenia, researchers may develop drugs with improved efficacy and fewer side effects [71].

## 10. Novel Approaches to Treat Psychosis and Schizophrenia: Targeting Neural Networks and Circuits

### Moving Beyond Single Neurotransmitters and Receptors

Traditional antipsychotic drugs primarily focus on modulating single neurotransmitters (e.g., dopamine) and receptors (e.g., D2 receptors) [72]. However, recent advances in neuroscience have revealed the complexity of neural networks and circuits involved in schizophrenia [73]. Rather than targeting isolated neurotransmitters, researchers are exploring interventions that modulate entire neural networks [74]. This holistic approach recognizes the interconnectedness of brain regions and their contributions to schizophrenia symptomatology [75]. By targeting specific circuits, researchers aim to achieve more precise and effective treatments.

## 11. Molecular Polypharmacy: One Drug, Multiple Targets—KarXT: A Promising Example

Molecular polypharmacy is an emerging concept in drug development, where a single drug targets multiple molecular pathways implicated in a complex disorder like schizophrenia [76,77]. One promising example is KarXT, a novel drug that simultaneously addresses psychosis and cognitive dysfunction [78]. KarXT combines xanomeline, which targets the muscarinic acetylcholine system, with trospium, an anticholinergic agent [79]. This combination demonstrates the potential of polypharmacy in one pill, providing a comprehensive approach to schizophrenia treatment [80]. By simultaneously addressing different aspects of the disorder, such as positive symptoms and cognitive deficits, KarXT represents a promising advancement in the development of drugs to treat psychosis and schizophrenia (as well as likely symptom domains and disorders beyond that involve presynaptic hyperdopaminergia and/or an imbalance between excitation and inhibition related to GABA and glutamate transmission and interaction [79,81,82].

## 12. Known and Emerging Molecular Targets for Specific Symptoms

### Positive Psychotic Symptoms

Positive psychotic symptoms, such as hallucinations and delusions, have long been associated with dysregulated dopamine transmission in the brain [83]. While this neurotransmitter–receptor dyad remains a critical target, researchers are exploring additional molecular targets to improve treatment efficacy and reduce side effects [84,85].

1. **Presynaptic vs. postsynaptic dopamine modulation:** Traditional drugs targeting psychosis and schizophrenia primarily act by blocking postsynaptic dopamine D2 receptors. However, the presynaptic modulation of dopamine release is also under investigation as a potential target to fine-tune dopamine transmission [86,87,88,89].
2. **GABA and glutamate systems:** The dysregulation of the gamma-aminobutyric acid (GABA) and glutamate systems has been implicated in schizophrenia. Novel drugs targeting these systems may offer alternative treatment options [90].
3. **Muscarinic acetylcholine system:** The muscarinic acetylcholine system plays a role in cognitive function. Drugs that modulate this system, such as xanomeline, are being explored to address cognitive deficits in schizophrenia [91,92].
4. **Trace amine-associated receptor 1 (TAAR1):** TAAR1 is a receptor involved in modulating dopamine and other neurotransmitters. Targeting TAAR1 may provide a unique approach to regulating neurotransmitter systems in schizophrenia [93].
5. **Excitation–inhibition imbalance:** Schizophrenia is characterized by an excitation–inhibition imbalance in neural circuits. Modulators that restore this balance are under investigation [94,95,96].

## 13. Molecular Underpinnings and Targets for Negative Symptoms

Negative symptoms in schizophrenia, including social withdrawal, anhedonia, and apathy, pose significant challenges in treatment. Identifying the molecular underpinnings and targets for these symptoms is crucial for improving the quality of life for affected individuals [97].

1. **Dopamine receptors:** While positive symptoms are associated with excess dopamine activity, negative symptoms may result from deficits in dopamine transmission. Balancing dopamine receptor activity is a potential approach [98].
2. **Glutamate and NMDA receptors:** The glutamate system, particularly N-methyl-D-aspartate (NMDA) receptors, has been implicated in negative symptoms. Enhancing NMDA receptor function is under investigation [99] (Table 1).
3. **Oxytocin receptors:** Oxytocin, a neuropeptide, has shown promise in alleviating negative symptoms [100]. Drugs targeting oxytocin receptors may improve social functioning [101].

## 14. Molecular Underpinnings and Targets for Cognitive Dysfunction

Cognitive dysfunction is a pervasive and debilitating aspect of schizophrenia. Impaired memory, executive function, and attention significantly impact daily functioning. Identifying the molecular underpinnings and targets for cognitive deficits is crucial for enhancing patients’ cognitive abilities [102].

1. **Glutamate and AMPA receptors:** Enhancing glutamate signaling, particularly through AMPA receptors, is a potential strategy to improve cognitive function [103] (Table 1).
2. **Cholinergic systems:** The cholinergic system, including nicotinergic and muscarinic receptors, plays a role in cognitive processes. Drugs targeting these receptors may enhance cognitive abilities [104].
3. **Neuroplasticity:** Promoting neuroplasticity, the brain’s ability to reorganize and adapt, is another avenue of research for addressing cognitive deficits in schizophrenia [105,106,107].

## 15. Molecular Underpinnings and Targets for Improved Reward System Functioning

The reward system in the brain is implicated in motivation, pleasure, and goal-directed behavior. The dysregulation of this system can lead to anhedonia and reduced motivation [108], common negative symptoms in schizophrenia [109,110,111]. Identifying molecular targets to restore normal reward system functioning is essential.

1. **Dopamine pathways:** Modulating dopamine pathways involved in reward processing may help restore motivation and pleasure in individuals with schizophrenia [112,113].
2. **Opioid receptors:** Opioid receptors are also involved in reward processing. Targeting these receptors may offer therapeutic potential [114].
3. **Serotonin receptors:** Serotonin receptors, particularly the 5-HT2A subtype, have been implicated in reward dysfunction and may be targeted to restore normal functioning [115] (Table 1).

## 16. Molecular Underpinnings and Targets for Improved Illness Insight

One of the challenges in schizophrenia treatment is anosognosia, a lack of insight into the illness. Individuals with schizophrenia may not recognize their symptoms or the need for treatment. Identifying molecular targets to improve illness insight is essential for enhancing treatment engagement [116].

1. **Dopamine and prefrontal cortex:** Dysregulated dopamine signaling in the prefrontal cortex is associated with impaired insight. Targeting this circuitry may help individuals gain a better awareness of their illness [117].
2. **Glutamate and cognitive functioning:** Improving cognitive function, particularly in areas related to self-awareness, may contribute to increased illness insight [118].
3. **Neuroplasticity:** Promoting neuroplasticity and cognitive flexibility may enhance individuals’ ability to understand and accept their condition [119].

## 17. Molecular Underpinnings and Targets of Treatment-Resistant Schizophrenia

Treatment-resistant schizophrenia (TRS) presents a formidable challenge [120,121,122]. Some individuals with schizophrenia do not respond adequately to available treatments [123,124,125,126,127]. Identifying the molecular underpinnings and targets specific to TRS is critical for developing interventions that can break through the treatment resistance barrier.

1. **Dopamine dysregulation:** While traditional drugs targeting psychosis and schizophrenia primarily target the dopamine system, novel approaches to modulate dopamine pathways are under investigation [9,128].
2. **Glutamate dysfunction:** Restoring glutamate balance, particularly through NMDA receptor modulation, is a focus in TRS research [129].
3. **Cholinergic systems:** The cholinergic system, particularly the muscarinic receptors, regulate GABA and glutamate as well as dopamine, and each may be involved in TRS. Drugs targeting these receptors may enhance cognitive abilities [87,130].
4. **Inflammatory mechanisms:** Inflammation in the brain has been implicated in TRS [131,132,133]. Targeting neuroinflammatory pathways may provide new treatment avenues [134] (Table 1).

## 18. Molecular Targets for Disease Modification

Beyond symptom management, there is a growing interest in developing treatments that modify the course of schizophrenia. These disease-modifying interventions aim to address the underlying pathophysiological processes and potentially slow down or halt disease progression.

1. **Synaptic pruning:** Excessive synaptic pruning during adolescence is believed to contribute to schizophrenia development. Interventions that regulate this process are under investigation [135].
2. **Neuroinflammation:** Chronic neuroinflammation is associated with schizophrenia. Modulating immune responses, microglial activation, and synaptic pruning in the brain may have disease-modifying effects [136] (Table 1).
3. **Neuroplasticity:** Promoting neuroplasticity and neural repair mechanisms may help mitigate the long-term consequences of schizophrenia [137].

## 19. Molecular Targets for Improved Safety and Tolerability

Currently, medications approved to treat schizophrenia often come with a range of side effects, including sedation/somnolence and agitation or insomnia, prolactin elevation and sexual dysfunction, neuromotor symptoms, and weight gain and metabolic disturbances [138,139,140]. Identifying molecular targets to avoid adverse effects and enhance the safety and tolerability of agents targeting psychosis and schizophrenia is crucial to improving treatment adherence and overall patient well-being. Balancing efficacy and effectiveness with safety and tolerability is essential. Strategies to reduce weight gain, dyslipidemia, and insulin resistance are actively being explored, as these are among the strongest contributors to the life-shortening features of schizophrenia [141,142] that are partly inherent to the illness, partly related to an unhealthy lifestyle, and partly related to adverse effects of treatments targeting schizophrenia [143].

1. **Dopamine receptor subtypes**: Selectively targeting specific dopamine receptor subtypes may reduce side effects while preserving efficacy for psychosis [144].
2. **Serotonin receptors**: Modulating serotonin receptors can influence side effect profiles. Balancing dopamine and serotonin interactions is a focus of research [145].
3. **Metabolic pathways**: Understanding the metabolic effects of drugs targeting psychosis and schizophrenia and developing interventions to mitigate these adverse effects are crucial. This can involve added molecular activity as part of a drug with efficacy for positive, negative, and/or cognitive symptoms or adding self-standing medications to currently approved antidopaminergic drugs and other treatments targeting psychosis, such as metformin of GLP-1 agonists [146,147].
4. **Neuroprotection**: Developing neuroprotective agents that shield the brain from the adverse effects of medications targeting psychosis and schizophrenia is a promising avenue. These agents may help prevent structural and functional changes associated with long-term antidopaminergic use or stimulate BDNF or other neurotrophic and antiapoptotic processes [148].

## 20. Combination Treatments to Minimize Side Effects

Combining multiple medications with complementary mechanisms of action is another strategy to minimize side effects while preserving therapeutic efficacy. This approach can help offset the adverse effects associated with individual drugs, resulting in a more tolerable treatment regimen.

1. **Olanzapine plus samidorphan:** Olanzapine is an effective medication for schizophrenia, but it is associated with weight gain. Samidorphan, an opioid receptor antagonist, is combined with olanzapine in an attempt to mitigate this side effect, providing a more tolerable treatment option [149,150].
2. **Xanomeline plus trospium:** A s previously mentioned, KarXT combines xanomeline, which targets the muscarinic acetylcholine system, with trospium, an anticholinergic agent. This combination minimizes the cholinergic side effects associated with muscarinic agonists [151,152].

These combination treatments exemplify the concept of molecular polypharmacy, where multiple drugs with distinct molecular targets work together synergistically to enhance therapeutic outcomes while minimizing side effects.

## 21. Novel Modes of Delivery for Medications Targeting Psychosis and Schizophrenia

In addition to innovative drug targets, researchers are exploring novel modes of drug delivery to improve the precision, convenience, and adherence of treatments targeting psychosis and schizophrenia. Some of these approaches include the following:

1. **Subcutaneous delivery:** Subcutaneous injections offer a more controlled and sustained release of medication compared to oral formulations. This mode of delivery can help minimize fluctuations in drug levels and improve adherence [153,154,155].
2. **Transdermal patches:** Transdermal patches provide a non-invasive and potentially convenient method of drug delivery. They can ensure the steady absorption of medication over an extended period, reducing the need for frequent dosing and improving the pharmacokinetic properties of some drugs [156,157].
3. **Intranasal administration:** Intranasal sprays and powders allow for rapid drug absorption through the nasal mucosa. This mode of delivery can lead to a faster onset of action, particularly important in managing acute psychotic episodes [158,159].
4. **Viral vector-based delivery:** Advanced techniques involving viral vectors can be used to target specific brain regions or neural circuits. This precision allows for localized drug delivery, minimizing systemic side effects [160,161,162,163].

These novel delivery methods hold promise in tailoring treatments to individual patient needs, enhancing treatment efficacy, and reducing the burden of daily medication regimens.

## 22. Pharmacokinetic Mechanisms to Speed Up or Extend Drug Effects

Pharmacokinetic strategies aim to optimize the absorption, distribution, metabolism, and elimination of drugs targeting psychosis and schizophrenia to achieve specific treatment goals. Depending on the clinical scenario, these mechanisms can either speed up drug delivery for rapid release and relief or extend the duration of drug effects to enhance adherence.

1. **Rapid-acting drugs for psychosis and schizophrenia:** For the management of acute agitation and psychosis, rapid-acting antipsychotic formulations are crucial. Short-acting intramuscular, inhalable, or intranasal administration can provide rapid relief within minutes [159,164,165].
2. **Long-acting gastrointestinal delivery:** Slowing down the intestinal transit of oral medications can potentially lead to a once-weekly oral depot whereby patients with difficulties with adherence would only require once weekly reminders or supervision [166].
3. **Long-acting injectable drugs for psychosis and schizophrenia:** To improve treatment adherence, long-acting injectable “antipsychotics” (LAIs) postsynaptic antidopaminergic medications offer extended drug release over weeks or months. LAIs ensure that patients receive consistent treatment without the need for daily dosing, reducing the risk of relapse. Administrations routes include intramuscular and subcutaneous administration [167,168].

## 23. Conclusions

Schizophrenia remains a complex and challenging mental disorder that affects millions of individuals worldwide. While postsynaptic antidopaminergic drugs have been the cornerstone of treatment for decades, there is still much to discover and improve upon in the field of schizophrenia therapeutics.

Advancements in understanding the molecular mechanisms underlying schizophrenia and the development of novel drug targets offer hope for more effective treatments. Targeting specific symptom domains, such as positive and negative symptoms, cognitive dysfunction, and impaired insight, allows for a more comprehensive approach to schizophrenia management.

Innovative strategies, such as molecular polypharmacy, combination treatments, and novel drug delivery methods, aim to improve treatment efficacy while minimizing side effects. Additionally, pharmacokinetic mechanisms can tailor drug effects to meet the unique needs of individual patients.

The ongoing pursuit of a better understanding of the molecular underpinnings of schizophrenia and the development of innovative treatment approaches provide hope for improved outcomes and a higher quality of life for individuals living with this challenging disorder.

## Figures and Tables

**Table 1:** Molecular mechanisms involved in schizophrenia.

Mechanism	Variation	Outcome
Dopamine Dysregulation	Hyperactivity in Mesolimbic Pathway	Positive Symptoms
	Hypoactivity in Mesocortical Pathway	Negative Symptoms
Glutamate Hypofunction	NMDA Receptor Dysfunction and Cortical Excitability	Cognitive Deficits and Negative Symptoms
Serotonin Imbalance	5-HT2A Receptor Overactivity and Dopamine Release	Positive and Negative Symptoms
Genetic Factors	DISC1, NRG1, and DTNBP1 Genes and Synaptic Function	
Neuroinflammation	Microglial Activation and Synaptic Pruning	

## Data Availability

No new data were created or analyzed in this study. Data sharing is not applicable to this article.
